# Commentary on “A strait or an ocean? Exploring risks and resources among people who use drugs in Denmark and Sweden”

**DOI:** 10.1177/14550725251396866

**Published:** 2025-11-21

**Authors:** Jenni Savonen

**Affiliations:** 1 Center of Law and Welfare, University of Eastern Finland, Joensuu, Finland

**Keywords:** attitudes, drug policy, Finland, harm-reduction, lay knowledge

In their article “A strait or an ocean? Exploring risks and resources among people who use drugs in Denmark and Sweden”, Houborg et al. (2025) contribute to the discussion of impacts of drug policy, specifically those that shape risk environments and the everyday lives of structurally vulnerable people who use hard drugs. The authors remind the reader that the results of the study are not generalizable or conclusive, but rather exploratory and descriptive. This is both reasonable and true yet does not take away from the lessons learned from the study.

It is impossible to imagine a research setting where drug policy and its effects could be isolated from cultural, social, economic and various other issues. This is why the design and results of this compelling study perhaps benefit from an evidence-based leap of faith to help dive into the comparison of two drug use contexts. The article's key argument – that drug policies affect risk environments and experiences of people who use drugs – is credible and supported by the results of this study, theory and previous research. The use of drug consumption rooms (DCRs) in Denmark compared to Sweden is an obvious example and is supported in a comparison study between 20 countries, where more liberal drug policies were reported to encourage the adoption of harm reduction strategies (Benfer et al., 2018).

However, the effects of policy are more pervasive than this. Drug policy not only governs responses to drug issues, but also creates realities. A society where drug use is criminalized creates an environment where a person who uses drugs is breaking the law and is viewed as immoral. These negative portrayals evoke stigmatization and marginalization. When drug use is not criminalized, the meanings given to a person who uses drugs are different; people are not criminals behaving against common understandings of right and wrong, but rather as people making rational choices or people with an addiction illness. This is essentially a difference between a criminal justice framework and a health framework (see also Unlu et al., 2021), which set the stage for lay understandings and attitudes towards the drug issue. These, in turn, are inevitably reflected in how the society and other people treat people who use drugs, which again affects their everyday lives and identities.

While drug policy creates lay understandings, these understandings also have an impact on how the drug issue is viewed and on the measures that are seen as appropriate in responding to it. Consequently, drug policy and lay understandings operate in a cycle. The “What's the problem represented to be?” approach (Bacchi, 2018) used in policy analysis delves into this dynamic: How we construct the “drug problem” works to justify acceptable responses to the problem, and therefore policy. Partly through political choices, these problematizations have discursive (how we talk about drug use), lived (what services are provided) and subjectification (how people who use drugs are positioned) effects. It is thus not only different policies, but also different realities and the consequences that are produced by them that contribute to the explanations of differences in Denmark and Sweden described in this study.

As another welfare state, Finland's drug policy falls closer to that of Sweden than that of Denmark. Finland also has a repressive drug policy where drug use is criminalized, although, following an increase in drug use in the mid-1990s, Finland adopted harm-reduction measures more vigorously than Sweden. Harm-reduction measures became firmly established alongside criminal control measures and needle exchange program, for example, are widely accepted by the general population today. However, attitudes towards new harm-reduction measures, such as DCRs, are more reluctant (Savonen et al., 2023). An initiative to open a DCR in the Helsinki metropolitan area was started already in 2018, but the process has not yet reached a point where this would be possible in practice. One key issue is that DCRs require a change in policy as at least a partial decriminalization of use (Unlu et al., 2021). Considering the arguments of Houborg et al. (2025), not having DCRs can be linked to risky drug using environments also in Finland because it has one of the highest rates of drug-related deaths in Europe.

Drug policy needs to consider both prevention goals and the needs of people who use drugs (Benfer et al., 2018). This is why studies focusing on people who use drugs are extremely relevant, especially with a volume such as that in the study by Houborg et al. (2025) (*N* = 474). Their article tells a hopeful story about the wide use of social and health care services in two countries with different drug policies, showing that the welfare states succeed in providing low-threshold services that reach structurally vulnerable people. There is still room to go deeper into the user-experiences of people within these services: What is the experience of using the services? Does it feel more difficult, stigmatizing or shameful for people in the context of stricter policy? What is the impact on wellbeing or identities? While drug policy and lay understandings of drug use pertain to all people in a society, their discursive, lived and subjectification consequences mainly fall on people who use drugs. This is a good reminder that is well illustrated by Houborg et al. (2025) in their study.
